# Right Atrial Mechanics in Healthy Mid-Term Pregnancy—An Analysis from a Three-Dimensional Speckle-Tracking Echocardiographic MAGYAR-Preg Study

**DOI:** 10.3390/biomedicines14061216

**Published:** 2026-05-28

**Authors:** Attila Nemes, Renáta Halcsik, Árpád Kormányos, Nándor Gyenes, Kitti Rajcsány, Barbara Bordács, Nóra Ambrus, Mohammad Nasiri, Csaba Lengyel, Tibor Novák

**Affiliations:** 1 Department of Medicine, Albert Szent-Györgyi Medical School, Albert Szent-Györgyi Clinical Center, University of Szeged, Semmelweis street 8, P.O. Box 427, H-6725 Szeged, Hungary; halcsik.renata@med.u-szeged.hu (R.H.); kormanyos.arpad@med.u-szeged.hu (Á.K.); gyenes.nandor@med.u-szeged.hu (N.G.); rajcsany.kitti@med.u-szeged.hu (K.R.); bordacs.barbara.aniko@med.u-szeged.hu (B.B.); ambrusnora@gmail.com (N.A.); lengyel.csaba@med.u-szeged.hu (C.L.); 2 Department of Obstetrics and Gynaecology, Albert Szent-Györgyi Medical School, University of Szeged, P.O. Box 427, H-6725 Szeged, Hungary; nasiri.mohammad@med.u-szeged.hu (M.N.); novak.tibor@med.u-szeged.hu (T.N.)

**Keywords:** healthy, pregnancy, right atrial, strain, three-dimensional, echocardiography

## Abstract

**Introduction.** Pregnancy is characterized by a significant expansion of plasma volume and an increase in cardiac output, necessitating structural and functional adaptations of the cardiac chambers, including the right atrium (RA). To evaluate these changes, three-dimensional (3D) speckle-tracking echocardiography (STE) was used as a validated and sophisticated modality for the concurrent assessment of RA volumetric and functional alterations. This study aimed to characterize RA volumes, volume-based functional indices, and strain parameters in healthy women during mid-gestation, compared with a cohort of non-pregnant controls. **Methods.** This retrospective cohort analysis included 20 healthy, asymptomatic women in their second trimester (mean age: 29.9 ± 3.0 years; weight: 81.2 ± 14.2 kg; height: 166.9 ± 5.8 cm; body surface area [BSA]: 1.95 ± 0.17 m^2^). The control group consisted of 30 age-matched healthy non-pregnant women (mean age: 29.9 ± 4.1 years; weight: 58.7 ± 6.5 kg; height: 166.0 ± 5.4 cm; BSA: 1.68 ± 0.11 m^2^). All subjects underwent comprehensive two-dimensional Doppler echocardiography and 3DSTE. **Results.** Early and late diastolic RA volumes were significantly reduced, despite preserved end-systolic RA volume. Pregnant subjects exhibited reduced active RA stroke volume and increased passive RA emptying fraction, while all other parameters remained comparable between the groups. No significant differences were observed between groups in end-systolic peak RA global or mean segmental strains, nor in RA strains measured during atrial contraction. However, end-systolic peak regional RA strain analysis revealed decreased basal RA circumferential strain (CS) and increased superior RA-CS in pregnant participants compared with controls. Furthermore, during late diastole (at atrial contraction), superior RA-CS, RA-3D strain, and RA-area strain were significantly higher in healthy pregnant subjects than in controls. **Conclusions.** Substantial regional changes in RA function were detected by 3DSTE, likely reflecting adaptation to pregnancy-induced plasma volume expansion, and resulting in significant RA volumetric changes.

## 1. Introduction

During pregnancy, plasma volume and cardiac output increase significantly, leading to major structural and functional changes in the cardiac chambers [1,2]. The right atrium (RA) plays a pivotal role in the physiological adaptation of the right heart and pulmonary circulation, exhibiting continuous modulation of its volume and myocardial contractility throughout the cardiac cycle [3]. Considering the profound cardiovascular changes inherent to gestation, it is essential to delineate alterations in RA morphology and function under healthy maternal and fetal conditions. To this end, three-dimensional (3D) speckle-tracking echocardiography (3DSTE) was employed as a validated and advanced imaging modality to concurrently assess both the volumetric and functional changes in the RA [4,5,6,7,8,9,10,11]. Given the limited data on pregnancy-related RA characteristics, the present study aimed to characterize RA volumes, volume-based functional indices, and strain parameters in healthy women during mid-gestation and to compare these findings with those of a non-pregnant female cohort.

## 2. Subjects and Methods

### 2.1. Study Population

This retrospective cohort analysis involved 20 healthy, asymptomatic women in their second trimester (mean age: 29.9 ± 3.0 years). The control group consisted of 30 age-matched healthy non-pregnant women (mean age: 29.9 ± 4.1 years). Body surface area is estimated using the Mosteller formula, defined as the square root of the product of weight (kg) and height (cm) divided by 3600 [12,13]. Exclusion criteria for all participants included smoking, medication use, regular athletic training, and any underlying medical conditions. All participants underwent comprehensive two-dimensional Doppler echocardiography and 3DSTE. This retrospective cohort study was conducted within the framework of the MAGYAR-Preg study (Motion Analysis of the heart and Great vessels bY three-dimensionAl speckle-tRacking echocardiography in Pregnancy), an ongoing research initiative at the University of Szeged aimed at characterizing pregnancy-induced myocardial and valvular adaptations using 3DSTE. The acronym ‘Magyar,’ refers to the Hungarian term for ‘Hungarian.’ The study protocol complies with the principles of the Declaration of Helsinki and was approved by the Institutional and Regional Human Biomedical Research Committee of the University of Szeged (Ref. Nos. 71/2011 and 145/2021). As of 2026, all participants provided written informed consent prior to inclusion.

### 2.2. Two-Dimensional Doppler Echocardiography

Comprehensive two-dimensional Doppler echocardiographic assessments were performed in all participants by experienced sonographers (Á.K, N.G. and K.R.). Imaging was conducted using a Toshiba Artida™ system (Toshiba Medical Systems, Tokyo, Japan, now Canon Medical Systems, Otawara, Japan) coupled with a PST-30BT (1–5 MHz) phased-array transducer (Toshiba Medical Systems, Tokyo, Japan, now Canon Medical Systems, Otawara, Japan). Chamber quantification was performed in accordance with current professional guidelines and standard clinical protocols. With participants in the left lateral decubitus position, echocardiographic imaging was performed using standard parasternal and apical windows. Left atrial (LA) and left ventricular (LV) quantification was conducted using apical four-chamber and two-chamber long-axis views, and LV ejection fraction (LVEF) was calculated using Simpson’s biplane method. Doppler imaging was used to identify valvular regurgitation or stenosis, and to quantify early and late diastolic mitral inflow velocities [12,14].

### 2.3. Three-Dimensional Speckle-Tracking Echocardiography

**Figure 1:**
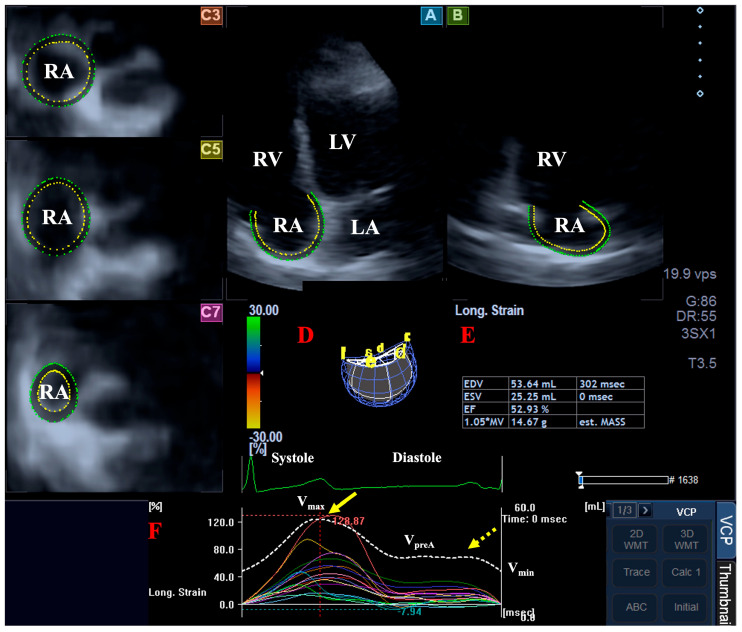
Illustrates the three-dimensional (3D) speckle-tracking echocardiographic (3DSTE) analysis of the right atrium (RA) in a representative subject using a full-volume dataset. The analysis integrates apical four-chamber (A) and two-chamber (B) long-axis views with short-axis views at the basal (C3), midatrial (C5), and superior (C7) levels to generate a comprehensive 3D RA model (D). The resulting output displays calculated RA volumetric data (E) and time-resolved curves for global RA volume changes (dashed line) and segmental RA longitudinal strains (colored lines) (F). The yellow arrow indicates peak RA strains, while the yellow dotted arrow marks RA strains specifically at the time of atrial contraction. Abbreviations: EDV, end-diastolic volume; ESV, end-systolic volume; EF, ejection fraction; Vmax, maximum RA volume; VpreA, pre-atrial contraction RA volume; Vmin, minimum RA volume; LA, left atrium; LV, left ventricle; RA, right atrium; RV, right ventricle.

3DSTE datasets were acquired using the same Toshiba Artida™ system, integrated with a PST-25SX matrix-array transducer (Toshiba Medical Systems, Tokyo, Japan, now Canon Medical Systems, Otawara, Japan). Volumetric 3D datasets were acquired digitally from the apical window during a single breath-hold. In subjects with sinus rhythm, six wedge-shaped subvolumes were acquired over six consecutive cardiac cycles to generate a full-volume 3D pyramidal dataset. Offline analysis was performed using 3D Wall Motion Tracking software (version 2.7, Toshiba Medical Systems, Tokyo, Japan, now Canon Medical Systems, Otawara, Japan). The 3D datasets were visualized in apical four-chamber and two-chamber long-axis views, along with three short-axis planes (basal, mid-atrial, and superior RA regions). Following the designation of reference points, the endocardial border was manually traced from the tricuspid annular plane, encompassing the RA, while excluding the RA appendage and caval veins. Automated 3D wall motion tracking was then applied from the end-diastolic reference frame, with manual refinements permitted as necessary (Figure 1) [4,5,6,7,8].

### 2.4. 3DSTE-Derived RA Quantifications

RA volumes were quantified at specific phases of the cardiac cycle using the aforementioned 3D model [9]:

- End-systolic maximum RA volume (at tricuspid valve opening, Vmax);
- RA volume at the onset of atrial contraction (corresponding to the P-wave on ECG, VpreA);
- End-diastolic minimum RA volume (at tricuspid valve closure, Vmin).

Based on these volumetric indices, the following RA functional parameters were derived [10]:

- Systolic reservoir function: represented by the total RA stroke volume (TASV = Vmax − Vmin) and the total RA emptying fraction (TAEF = TASV/Vmax).
- Early diastolic conduit function: characterized by the passive RA stroke volume (PASV = Vmax − VpreA) and the passive RA emptying fraction (PAEF = PASV/Vmax).
- Late diastolic booster pump function: defined by the active RA stroke volume (AASV = VpreA − Vmin) and the active RA emptying fraction (AAEF = AASV/VpreA).

Using the same 3D virtual model, several strain parameters were automatically computed, including unidirectional/unidimensional radial (RS), longitudinal (LS), and circumferential (CS) RA strains, reflecting segmental RA thinning/thickening, lengthening/shortening, and widening/narrowing of the RA segments, respectively. Additionally, multidimensional complex area strain and 3D strain were calculated; area strain reflects the combination of LS and CS, whereas 3D strain encompasses RS, LS, and CS. For a comprehensive evaluation, regional strains were derived from segmental values. 3DSTE-derived RA strain curves exhibited two distinct peaks: the first corresponding to end-systolic reservoir function, and the second reflecting end-diastolic atrial contraction (RA systole, booster pump function) [10].

### 2.5. Statistical Analysis

Continuous variables are reported as mean ± standard deviation, while categorical variables are expressed as absolute frequencies and percentages, as appropriate. Statistical significance was predefined at a *p*-value < 0.05. Normality was assessed using the Shapiro–Wilk test, and homogeneity of variances was evaluated with Levene’s test. For normally distributed variables, comparisons were performed using the independent samples Student’s *t*-test; otherwise, the Mann–Whitney U test was applied. Categorical variables were compared using Fisher’s exact test. Intraobserver and interobserver variability was assessed using the Bland–Altman method. A post hoc power analysis was conducted to evaluate the robustness of the findings. With the current sample size, a large effect size (Cohen’s d = 0.8) was observed, with a statistical power of 0.72 at an alpha level of 0.05. All statistical analyses were performed using SPSS software (version 22.0; IBM Corp., Armonk, NY, USA).

## 3. Results

### 3.1. Clinical and Two-Dimensional Doppler Echocardiography

Healthy, asymptomatic women in their second trimester had significantly higher body weight and body surface area compared to non-pregnant controls, while height was similar between the two groups (weight: 81.2 ± 14.2 kg vs. 58.7 ± 6.5 kg, *p* < 0.05; height: 166.9 ± 5.8 cm vs. 166.0 ± 5.4 cm, *p* = ns; body surface area: 1.95 ± 0.17 m^2^ vs. 1.68 ± 0.11 m^2^, *p* < 0.05). Healthy pregnant subjects exhibited a thicker interventricular septum, increased left ventricular ejection fraction, and reduced early transmitral flow velocities compared to non-pregnant individuals (Table 1). Notably, none of the participants had valvular regurgitation (≥grade 1) or significant valvular stenosis.

### 3.2. 3DSTE-Derived RA Volumes

Early diastolic VpreA and indexed-VpreA, as well as late diastolic indexed-Vmin, were significantly reduced, despite preserved end-systolic Vmax and indexed-Vmax. Regarding stroke volumes and emptying fractions, pregnant subjects exhibited decreased AASV and increased PAEF, while all other parameters remained comparable between the groups (Table 2).

### 3.3. 3DSTE-Derived RA Strains

No significant differences were observed between groups regarding end-systolic peak RA global and mean segmental strains, nor in RA strains measured at atrial contraction. However, end-systolic peak regional RA strain analysis revealed decreased basal RA-CS and increased superior RA-CS in healthy pregnant participants compared with controls. Furthermore, during late diastole (atrial contraction), superior RA-CS, RA-3D strain, and RA-area strain were significantly higher in healthy pregnant subjects compared with controls (Table 3 and Table 4).

## 4. Discussion

To accommodate maternal and fetal metabolic demands, pregnancy induces profound hemodynamic adaptations, marked by a 40–50% expansion in both plasma volume and cardiac output by the 32nd gestational week. This increase in cardiac output is initially driven by augmented stroke volume, whereas increased heart rate becomes the predominant contributor in later stages. Despite significant structural cardiac remodeling, myocardial function typically remains preserved. Key systemic features include reduced systemic vascular resistance and a shift toward a hypercoagulable state [1,2].

Although 3D echocardiography is a well-established modality for quantifying RA volumes, the evolving field of 3DSTE enables the simultaneous assessment of volumes, volume-based functional indices, and strain parameters from a single dataset using a reconstructed virtual 3D RA cast [4,5,6,7,8]. 3DSTE-derived normal reference ranges for RA volumes and strains are now available for clinical use [9,10]. As a non-invasive, efficient, and cost-effective tool—independent of contrast agents or ionizing radiation—3DSTE facilitates a comprehensive analysis of the three distinct phases of RA function: the reservoir phase during ventricular systole, the conduit phase during early diastole, and the booster pump phase during late diastole [3,4,5,6,7,8,9,10,11]. While the left atrial abnormalities accompanying pregnancy have been extensively investigated [15], available data suggest that numerous differences may be identified between the volumetric and functional parameters of the RA and left atrium, even regarding their adaptive responses to a given stimulus [16].

According to the literature, RA diameter and volume increase progressively from the first through the third trimester; however, this increase reaches statistical significance only when comparing the third trimester with the first. Postpartum, these values tended to decline [17]. Similarly, the RA area has been shown to increase during pregnancy [18]. This increase was significant in the 2nd trimester as compared with the 1st trimester; no further increase was seen in the 3rd trimester and the RA area reduced postpartum [15]. A significant expansion in both RA area and volume can be detected between the first and third trimesters. Following delivery in healthy subjects, the RA area and volume begin to decrease as early as one month postpartum, with a significant reduction observed by six months [19].

Regarding RA functions, previous studies have shown that global peak RA-LS, reflecting RA reservoir function and assessed by two-dimensional STE, increases significantly during pregnancy and normalizes during the postpartum period. Similarly, global peak RA contractility strain, an indicator of RA contraction, has been reported to increase during pregnancy and return to baseline after delivery [18]. In another study, no significant change was found in RA strain during pregnancy, while RA strain rates decreased throughout pregnancy, and reached their lowest values postpartum [15]. In contrast, systolic RA strain rate has been reported to increase progressively throughout pregnancy and into the postpartum period [20]. In another study, a significant decline in RA strain from the 1st to the 3rd trimester has been described in healthy individuals, followed by a marked recovery of RA strain one month postpartum [19]. Early diastolic RA strain rate decreases during pregnancy and partially recovers postpartum, whereas late diastolic RA strain rate shows only a mild reduction postpartum compared with baseline [20]. In Asian cohorts of healthy pregnant women, RA mechanics in the third trimester have been reported to be enhanced as compared to those of Caucasian cohorts, with higher reservoir and contractile strain values, suggesting improved RA compliance and contractile performance [21].

The present study has several important implications. First, our findings confirm that 3DSTE is a suitable method for the simultaneous volumetric and functional strain-based analysis of the RA. This capability supports its use in pathophysiological investigations, such as the present study. Second, despite preserved end-systolic RA volume (Vmax), reduced basal RA-CS and increased superior RA-CS were observed, indicating a significant regional adaptation in RA contraction-relaxation patterns. Third, decreased RA volume during early diastole (VpreA) was associated with increased passive emptying fraction (PAEF), suggesting that a greater proportion of blood transits through the RA during early diastole, possibly reflecting accelerated circulation. Fourth, decreased RA minimum volume (Vmin) and active stroke volume (AASV) during late diastole were accompanied by apparent hyperfunction of the superior RA region, as reflected by increased RA-CS, RA-3D strain, and RA-area strain values during atrial contraction.

These findings may be explained by several physiological factors. The second trimester corresponds to the nadir of systemic vascular resistance, during which significant vasodilation leads to a ‘volume shift’ toward the periphery. This may transiently reduce RA preload and consequently decrease RA wall stretch during the filling phase compared with the non-pregnant state or the third trimester. In addition, from the second trimester onward, the enlarging uterus in the supine position may partially compress the inferior vena cava, thereby reducing venous return to the RA. Furthermore, pregnancy-related hormonal changes, together with increased heart rate and sympathetic activity, may enhance RA contractility, promoting more efficient emptying into the right ventricle, and resulting in lower residual volumes at specific phases of the cardiac cycle. Finally, certain technical considerations should be acknowledged. When echocardiographic examinations are performed in the supine position, reduced venous return may result in relative RA underfilling, potentially leading to underestimation of the true atrial volumes [22]. The role of indexing should also be considered; however, in the present study, differences in RA volumes remained non-significant even after normalization. Overall, these findings highlight that the physiological alterations associated with pregnancy remain incompletely understood, and further investigation into the underlying mechanisms is warranted.

### Limitation Section

Several limitations of the present study warrant consideration:

- First, the image quality inherent to 3DSTE is generally lower than that of conventional two-dimensional echocardiography, which may impact data analysis [4,5,6,7,8,9,10,11].
- The assignment of the atrial septum to either atrium remains debated; in this research, the atrial septum was considered part of the RA during the creation of a virtual model of the RA.
- This research was not designed as a validation study, as the clinical validity of 3DSTE-derived atrial volumes and functional indices has already been established [23,24]. Consequently, direct comparisons with other imaging modalities—such as two-dimensional STE, cardiac magnetic resonance, or computed tomography—were intentionally omitted.
- The scope of the analysis was strictly confined to the RA; thus, volumetric and functional parameters of other cardiac chambers were not evaluated.
- Finally, the relatively modest sample size of the pregnant cohort may limit the statistical power to detect subtle differences in RA morphology and function. Despite the high reproducibility of our measurements, the potential for Type II errors cannot be entirely excluded.

## 5. Conclusions

Substantial regional changes in RA function were detected by 3DSTE, likely reflecting adaptation to pregnancy-induced plasma volume expansion, and resulting in significant RA volumetric changes.

## Figures and Tables

**Table 1:** Two-dimensional echocardiographic data of pregnant versus non-pregnant healthy subjects.

	Non-Pregnant Healthy Subjects (*n* = 30)	Pregnant Healthy Subjects (*n* = 20)
LA diameter (mm)	34.4 ± 4.0	33.0 ± 3.3
LV end-diastolic diameter (mm)	46.2 ± 3.3	44.6 ± 3.7
LV end-diastolic volume (mL)	92.5 ± 15.7	99.6 ± 17.9
LV end-systolic diameter (mm)	33.1 ± 11.0	28.3 ± 3.6
LV end-systolic volume (mL)	33.6 ± 8.1	30.2 ± 7.7 *
Interventricular septum (mm)	8.3 ± 1.2	9.0 ± 0.6 *
LV posterior wall (mm)	8.7 ± 1.6	8.9 ± 0.7
LV ejection fraction (%)	64.0 ± 4.0	71.0 ± 7.2 *
E velocity (cm/s)	88.2 ± 14.4	61.1 ± 16.9 *
A velocity (cm/s)	57.9 ± 11.8	51.9 ± 11.2

* *p* < 0.05 versus non-pregnant healthy cases. Abbreviations: LA = left atrium; LV = left ventricle; E and A = early and late diastolic transmitral inflow velocities.

**Table 2:** Comparison of three-dimensional speckle-tracking echocardiography-derived volumetric right atrial parameters between pregnant and non-pregnant healthy subjects.

	Non-Pregnant Healthy Subjects (*n* = 30)	Pregnant Healthy Subjects (*n* = 20)
Calculated Volumes		
V_max_ (mL)	42.2 ± 15.9	36.4 ± 7.5
V_max_/BSA (mL/m^2^)	23.4 ± 10.3	19.3 ± 3.5
V_pre_A (mL)	28.9 ± 11.4	22.0 ± 5.3 *
V_pre_A/BSA (mL/m^2^)	16.2 ± 7.3	11.0 ± 1.7 *
V_min_ (mL)	22.5 ± 10.0	18.4 ± 5.2
V_min_/BSA (mL/m^2^)	12.6 ± 6.1	9.0 ± 1.5 *
Stroke Volumes		
TASV (mL)	19.7 ± 10.1	18.0 ± 6.3
PASV (mL)	13.3 ± 7.7	14.4 ± 5.5
AASV (mL)	6.4 ± 4.9	3.6 ± 2.0 *
Emptying fractions		
TAEF (%)	46.3 ± 13.9	48.9 ± 10.7
PAEF (%)	30.9 ± 11.7	38.8 ± 10.2 *
AAEF (%)	22.4 ± 14.0	16.7 ± 8.9

* *p* < 0.05 versus non-pregnant healthy cases. Abbreviations: AASV = active right atrial stroke volume; AAEF = active right atrial emptying fraction; BSA = body surface area; PAEF = passive right atrial emptying fraction; PASV = passive right atrial stroke volume; TAEF = total right atrial emptying fraction; TASV = total right atrial stroke volume; V_max_ = maximum right atrial volume; V_min_ = minimum right atrial volume; V_pre_A = right atrial volume before atrial contraction.

**Table 3:** Comparison of three-dimensional speckle-tracking echocardiography-derived regional peak right atrial strain parameters between pregnant and non-pregnant healthy subjects.

	Non-Pregnant Healthy Subjects (*n* = 30)	Pregnant Healthy Subjects (*n* = 20)
Global		
RS (%)	−13.1 ± 7.0	−9.8 ± 7.9
CS (%)	22.6 ± 16.4	25.3 ± 9.4
LS (%)	41.2 ± 16.5	40.9 ± 16.6
3DS (%)	−4.7 ± 3.6	−4.7 ± 5.1
AS (%)	73.0 ± 47.0	79.0 ± 32.5
Mean segmental		
RS (%)	−18.4 ± 6.3	−15.8 ± 6.9
CS (%)	28.5 ± 15.6	30.2 ± 9.1
LS (%)	44.9 ± 15.6	43.3 ± 15.7
3DS (%)	−10.1 ± 4.5	−9.6 ± 5.4
AS (%)	81.2 ± 46.8	85.2 ± 31.5
Regional		
RS _basal_ (%)	−15.9 ± 6.2	−12.9 ± 6.4
RS _midatrial_ (%)	−19.1 ± 6.4	−15.5 ± 7.4
RS _superior_ (%)	−21.0 ± 14.4	−20.5 ± 11.9
CS _basal_ (%)	26.9 ± 13.2	18.6 ± 6.9 *
CS _midatrial_ (%)	24.5 ± 13.7	25.1 ± 8.2
CS _superior_ (%)	36.6 ± 31.6	55.0 ± 27.4 *
LS _basal_ (%)	49.8 ± 18.6	46.4 ± 20.7
LS _midatrial_ %)	52.1 ± 21.9	47.0 ± 22.6
LS _superior_ (%)	27.0 ± 20.3	33.1 ± 15.4
3DS _basal_ (%)	−9.0 ± 4.7	−7.6 ± 4.4
3DS _midatrial_ (%)	−9.8 ± 4.6	−8.1 ± 5.5
3DS _superior_ (%)	−12.4 ± 10.3	−14.7 ± 10.3
AS _basal_ (%)	71.8 ± 32.0	59.5 ± 30.8
AS _midatrial_ (%)	86.3 ± 51.7	80.0 ± 35.3
AS _superior_ (%)	87.3 ± 96.3	129.9 ± 77.6

* *p* < 0.05 versus non-pregnant healthy cases. Abbreviations: RS = radial strain; CS = circumferential strain; LS = longitudinal strain; 3DS = three-dimensional strain; AS = area strain.

**Table 4:** Comparison of three-dimensional speckle-tracking echocardiography-derived right atrial strain parameters at atrial contraction between pregnant and non-pregnant healthy subjects.

	Non-Pregnant Healthy Subjects (*n* = 30)	Pregnant Healthy Subjects (*n* = 20)
Global		
RS (%)	−6.3 ± 6.1	−6.5 ± 6.2
CS (%)	8.1 ± 7.9	11.2 ± 9.4
LS (%)	9.1 ± 9.1	8.3 ± 5.7
3DS (%)	−3.2 ± 5.2	−3.1 ± 4.7
AS (%)	18.6 ± 18.5	22.6 ± 19.6
Mean segmental		
RS (%)	−7.8 ± 5.3	−8.5 ± 5.2
CS (%)	11.1 ± 7.4	14.3 ± 6.9
LS (%)	11.5 ± 6.5	10.7 ± 5.1
3DS (%)	−4.5 ± 4.4	−5.7 ± 4.4
AS (%)	24.4 ± 17.8	28.9 ± 14.7
Regional		
RS _basal_ (%)	−6.8 ± 5.9	−6.3 ± 4.8
RS _midatrial_ (%)	−8.2 ± 6.0	−8.2 ± 5.4
RS _superior_ (%)	−8.7 ± 6.7	−12.2 ± 8.1
CS _basal_ (%)	9.8 ± 5.3	9.4 ± 6.0
CS _midatrial_ (%)	9.2 ± 6.6	10.7 ± 5.4
CS _superior_ (%)	15.8 ± 17.2	27.5 ± 21.1 *
LS _basal_ (%)	11.0 ± 8.1	10.1 ± 6.3
LS _midatrial_ (%)	13.2 ± 9.1	9.0 ± 6.2
LS _superior_ (%)	9.9 ± 7.8	14.1 ± 9.9
3DS _basal_ (%)	−3.9 ± 5.5	−3.7 ± 3.4
3DS _midatrial_ (%)	−4.7 ± 4.6	−4.1 ± 4.1
3DS _superior_ %)	−5.1 ± 5.5	−10.0 ± 8.4 *
AS _basal_ (%)	20.8 ± 16.7	19.7 ± 11.0
AS _midatrial_ (%)	24.2 ± 17.3	22.2 ± 14.7
AS _superior_ (%)	30.1 ± 34.3	53.1 ± 47.1 *

* *p* < 0.05 versus non-pregnant healthy cases. Abbreviations: RS = radial strain; CS = circumferential strain; LS = longitudinal strain; 3DS = three-dimensional strain; AS = area strain.

## Data Availability

The data presented in this study are available on request from the corresponding author. The data are not publicly available due to local restrictions.
